# Case Report: Long-Term Suppression of Paroxysmal Kinesigenic Dyskinesia After Bilateral Thalamotomy

**DOI:** 10.3389/fneur.2021.789468

**Published:** 2021-12-03

**Authors:** Masato Murakami, Shiro Horisawa, Kenko Azuma, Hiroyuki Akagawa, Taku Nonaka, Takakazu Kawamata, Takaomi Taira

**Affiliations:** ^1^Department of Neurosurgery, Tokyo Women's Medical University, Tokyo, Japan; ^2^Institute for Integrated Medical Sciences, Tokyo Women's Medical University, Tokyo, Japan

**Keywords:** paroxysmal kinesigenic dyskinesia, dystonia, antiepileptic drugs, remission, ventro-oral thalamotomy

## Abstract

**Background:** Paroxysmal kinesigenic dyskinesia (PKD) is a movement disorder characterized by transient dyskinetic movements, including dystonia, chorea, or both, triggered by sudden voluntary movements. Carbamazepine and other antiepileptic drugs (AEDs) are widely used in the treatment of PKD, and they provide complete remission in 80–90% of medically treated patients. However, the adverse effects of AEDs include drowsiness and dizziness, which interfere with patients' daily lives. For those with poor compatibility with AEDs, other treatment approaches are warranted.

**Case Report:** A 19-year-old man presented to our institute with right hand and foot dyskinesia. He had a significant family history of PKD; his uncle, grandfather, and grandfather's brother had PKD. The patient first experienced paroxysmal involuntary left hand and toe flexion with left forearm pronation triggered by sudden voluntary movements at the age of 14. Carbamazepine (100 mg/day) was prescribed, which led to a significant reduction in the frequency of attacks. However, carbamazepine induced drowsiness, which significantly interfered with his daily life, especially school life. He underwent right-sided ventro-oral (Vo) thalamotomy at the age of 15, which resulted in complete resolution of PKD attacks immediately after the surgery. Four months after the thalamotomy, he developed right elbow, hand, and toe flexion. He underwent left-sided Vo thalamotomy at the age of 19. Immediately after the surgery, the PKD attacks resolved completely. However, mild dysarthria developed, which spontaneously resolved within three months. Left-sided PKD attacks never developed six years after the right Vo thalamotomy, and right-sided PKD attacks never developed two years after the left Vo thalamotomy without medication.

**Conclusion:** The present case showed long-term suppression of bilateral PKDs after bilateral thalamotomy, which led to drug-free conditions.

## Introduction

Paroxysmal kinesigenic dyskinesia (PKD) is an autosomal dominant movement disorder characterized by transient dyskinetic movements, including dystonia, chorea, or both, triggered by sudden voluntary movements (1). PKD develops around the age of 10, and 32% of patients with PKD experience spontaneous remission without any treatment by the age of 20 (2). Additionally, 50–60% of patients with PKD reported a decrease in the frequency of attacks after the age of 20 (2, 3). Carbamazepine and other antiepileptic drugs (AEDs) are widely used in the treatment of PKD, and they provide complete remission in 80–90% of medically treated patients (1, 2, 4). However, the adverse effects of AEDs include drowsiness and dizziness, which interfere with the patients' daily lives (5). For those with poor compatibility with AEDs, other treatment approaches are warranted.

Our previous report showed complete remission of PKD in four family members treated by ablation of the ventro-oral (Vo) nucleus of the thalamus (Vo thalamotomy) (6). Herein, we report a case of long-term complete remission of bilateral PKDs after staged bilateral Vo thalamotomy. To our knowledge, this is the first report of a successful outcome of bilateral Vo thalamotomy for PKD.

## Case Report

**Figure 1:**
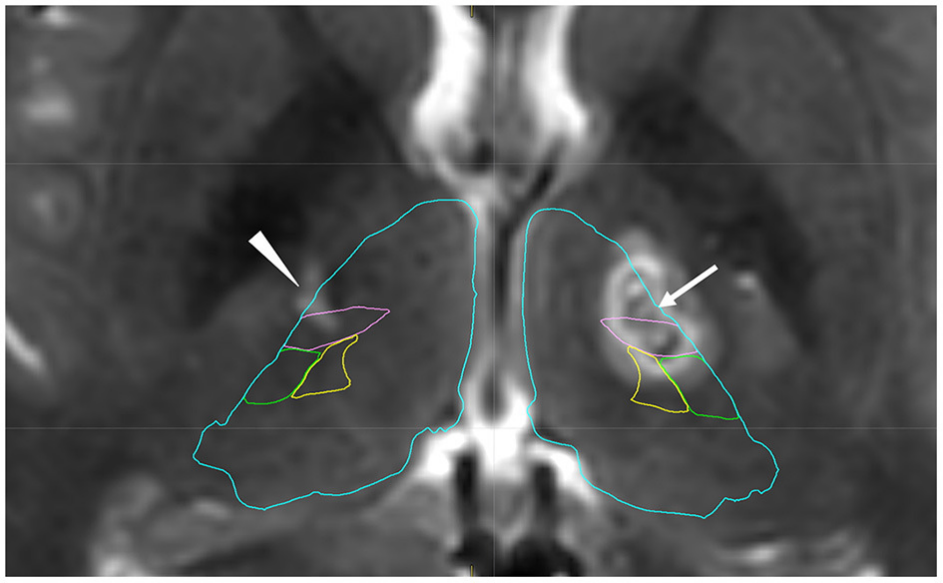
Postoperative T2-weighted image after left thalamotomy with anatomical mapping by Brainlab Elements. The arrow shows coagulated lesions in the left ventro-oral (Vo) nucleus. Posterior coagulated lesions are located in the ventral intermediate nucleus (Vim). The arrowhead shows an old lesion after previous surgery, which was confirmed in the Vo and Vim nucleus. Blue: thalamus, Pink: Vim, Green: ventral posterior lateral nucleus, Yellow: ventral posterior medial nucleus.

**Figure 2:**
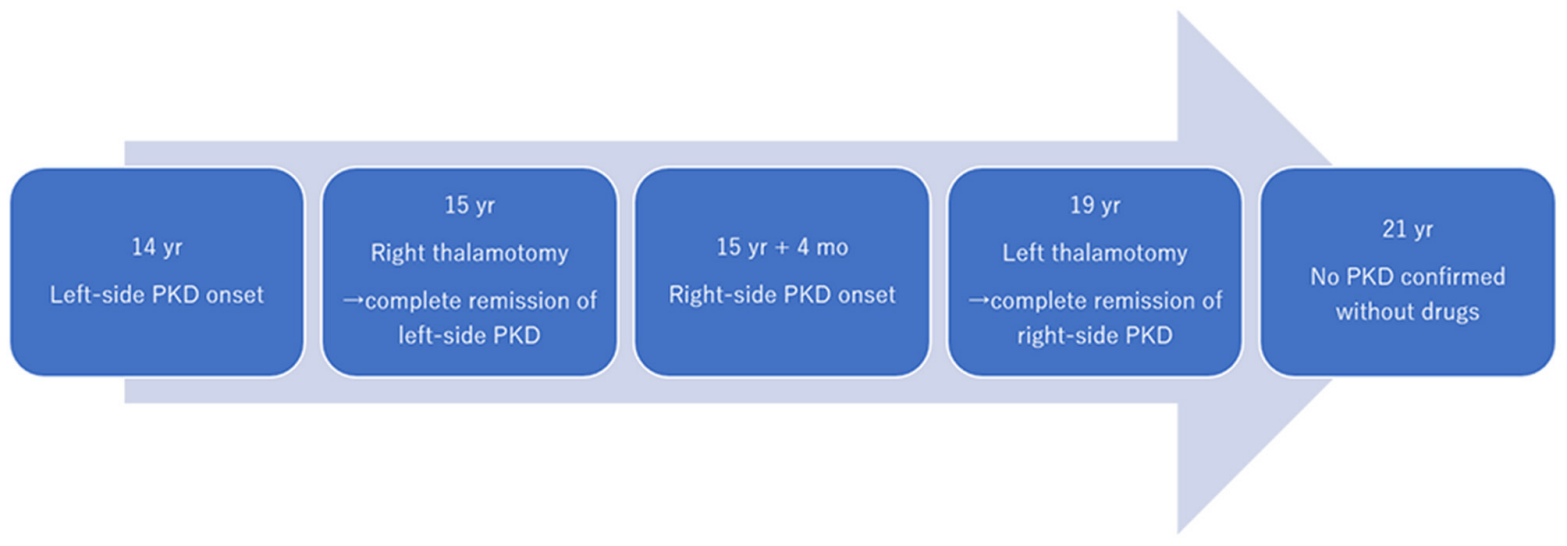
Time course of symptoms and interventions. PKD, paroxysmal kinesigenic dyskinesia; yrs, years; mo, month.

A 19-year-old man presented to our institute with right hand and foot dyskinesia. He had a significant family history of PKD. His uncle, grandfather, and grandfather's brother had involuntary movements triggered by voluntary movements. Proline-rich transmembrane protein 2 (PRRT2) on chromosome 16, which is associated with movement disorders including PKD (PRRT2-PxMD), was confirmed in his uncle and grandfather through genetic testing (c.649delC/pArg217-Glufs). All of them underwent stereotactic ablative surgery (Vo thalamotomy). The patient's grandfather and grandfather's brother experienced complete remission of involuntary movement attacks after the surgery. His uncle experienced a significant reduction in the frequency of daily attacks without medication. The patient first experienced paroxysmal involuntary left hand and toe flexion with left forearm pronation triggered by sudden voluntary movements at the age of 14. The frequency of attacks was 20–30 per day. Carbamazepine (100 mg/day) was prescribed, which led to a significant reduction in the frequency of attacks. However, drowsiness was significant after the intake of carbamazepine and significantly interfered with his daily life, especially school life. He underwent right-sided Vo thalamotomy at the age of 15, which resulted in complete resolution of PKD attacks immediately after the surgery. The detailed clinical course of right-sided Vo thalamotomy has been reported previously (6). Four months after the thalamotomy, the patient developed right elbow, hand, and toe flexion (Supplementary Movie 1). The frequency of attacks was 10–20 per day. The duration of the attacks ranged from 10 to 20 seconds. Carbamazepine (100 mg/day) was prescribed again, and the PKD attacks reduced to 5–10 times per day. However, the drowsiness induced by carbamazepine severely interfered with his daily and school lives. He underwent left-sided Vo thalamotomy at the age of 19. The target coordinate of the left Vo nucleus was set the same as in the previous surgery (15-mm lateral, 2-mm posterior, and 1-mm superior to the midpoint of the anterior commissure-posterior commissure). A total of six lesions were created on the left Vo nucleus in the same manner as in the previous surgery. Immediately after surgery, the PKD attacks resolved completely. However, mild dysarthria developed, which spontaneously resolved within three months. Left-sided PKD attacks never developed six years after the right Vo thalamotomy, and right-sided PKD attacks never developed two years after the left Vo thalamotomy without medication. The locations of the coagulated lesions were confirmed using Brainlab Elements. Bilateral lesions covered the Vo and ventral intermediate (Vim) nuclei (Figure 1). The Vo nucleus is located just anterior to the Vim nucleus of the thalamus. We included both anterior and posterior coagulated lesions in order to cover the entire Vo nucleus. Based on our experience, insufficient lesions to cover the Vo nucleus are likely to develop symptomatic recurrence. The time course of PKD onset and intervention is shown in Figure 2. The data for this study were retrospectively collected and analyzed. Considering the observational nature of the study, the ethics committee of our institution approved this study, and patient consent was waived.

## Discussion

To our knowledge, this is the first report of long-term complete resolution of PKD treated by staged bilateral thalamotomy. Transient mild dysarthria developed after the second thalamotomy. No other neurological deficits were observed. Currently, the patient is free from PKD attacks and oral medications.

The pathophysiology of PKD remains unknown. However, recent neuroimaging studies have suggested that the disruption of both structural and/or functional properties in the basal ganglia-thalamocortical circuitry is associated with PKD. The gray matter volume in the pre-supplementary motor area and right opercular part of the inferior frontal gyrus was reduced in patients with PKD when compared to healthy controls (7). Thalamic involvement in PKD was suggested by thalamic abnormalities such as volume reduction, regional shape deformation, and increased functional anisotropy in patients with PKD (8). Resting state functional MRI revealed that patients with PKD had increased functional and structural connectivity between ventral lateral/anterior thalamic nuclei and a lateral motor area when compared to controls (9). The ventral lateral nucleus of the thalamus, which consists of the Vo and Vim nucleus, is a motor thalamic nucleus. The motor thalamus plays a central role in movement control because it receives projections from the deep cerebellar nuclei and basal ganglia, including the substantia nigra pars reticulata and the internal segment of the globus pallidus (10). The Vo nucleus is a surgical target for ablation or stimulation in the treatment of task-specific focal dystonia, which manifests dystonic symptoms triggered by specific movements (11–14). In terms of the pattern of symptomatic manifestation, task-specific focal dystonia and PKD, both of which are triggered by movements, may share common neural mechanisms. Postoperative MRI in the present case showed a posterior lesion covering the Vim nucleus. It is still uncertain whether the Vo nucleus, Vim nucleus, or both were involved in the resolution of PKD in this case.

Bilateral thalamotomy is generally notencouraged due to irreversible complications such as dysarthria, dysphonia, and dysphagia, which develop in 20–30% of patients (15–18). However, majority of the available data for bilateral thalamotomy were published from the 1960s to 1980s when MRI and CT were not generally available to plan and confirm the ablative targets. Recent studies of bilateral thalamotomy indicated that dysarthria was mild or transient (19–24). Nevertheless, the second thalamotomy should be carefully considered, and for a safe second thalamotomy, it is recommended to verify the absence of adverse events associated with the first thalamotomy, keep the longest possible interval between the first and second surgery (at least 12 moths), include patients aged less than 70 years, and ensure the smallest possible lesion volume. However, the safety of bilateral thalamotomy has not been validated, and it is undeniable that serious complications including speech, swallowing, and balance problems can occur. In view of avoiding the possible complications associated with bilateral thalamotomy, deep brain stimulation (DBS) is highly expected to be an alternative surgical treatment option which can provide similar benefits to those of thalamotomy without permanent lesions. Complete suppression of paroxysmal non-kinesigenic dyskinesia after pallidal DBS has been reported (25). Vo-DBS is reported to be effective for focal hand dystonia (12, 13). Thus, pallidal or Vo-DBS may be useful for the treatment of PKD.

AEDs, including carbamazepine, phenytoin, and sodium valproate, are used to treat PKD and can provide complete remission in more than 85% of patients (2). The most widely available AED is carbamazepine, which is effective at a relatively low dose of 50–200 mg/day. However, even with a low-dose of carbamazepine (100 mg), the present case experienced relatively severe drowsiness, resulting in discontinuation of the drug. Spontaneous remission of PKD is expected in 32% of patients with PKD until the age of 20 (2). After the age of 20, 53.7% of patients reported a decrease in attack frequency (2). Thus, approximately half of the patients with PKD may suffer for several decades, necessitating long-term intake of AEDs. Long-term treatment with AEDs may result in bone abnormalities, sexual dysfunction, and reproductive disorders (5, 26, 27). Additionally, Stevens-Johnson syndrome and toxic epidermal necrolysis are rare and fatal skin reactions that are most commonly precipitated by AEDs (28). The present patient's uncle had been on AEDs for PKD until the age of 39, when he underwent thalamotomy. Some patients have had PKD symptoms for several decades. Our experiences, including previous reports, indicate that thalamotomy could result in patients with PKD being completely drug free. For those who are reluctant to continue AEDs due to adverse effects, thalamotomy can be an alternative treatment option. Larger sample sizes are needed to elaborate on the efficacy and safety of thalamotomy for PKD. The present case showed long-term suppression of bilateral PKDs after bilateral thalamotomy, which led to drug-free conditions.

## Data Availability Statement

The original contributions presented in the study are included in the article/Supplementary Material, further inquiries can be directed to the corresponding author.

## Ethics Statement

The studies involving human participants were reviewed and approved by The Ethics Committee of Tokyo Women's Medical University. The ethics committee waived the requirement of written informed consent for participation.

## Author Contributions

MM, HA, and KA: Execution. SH: Conception, organization, execution, and writing of the draft and figure. TK: Organization. TT: Conception, organization, and execution. All authors contributed to the manuscript and approved the submitted version.

## Funding

This work was supported by the Takeda Science Foundation and Grant-in-Aid for Scientific Research.

## Conflict of Interest

The authors declare that the research was conducted in the absence of any commercial or financial relationships that could be construed as a potential conflict of interest.

## Publisher's Note

All claims expressed in this article are solely those of the authors and do not necessarily represent those of their affiliated organizations, or those of the publisher, the editors and the reviewers. Any product that may be evaluated in this article, or claim that may be made by its manufacturer, is not guaranteed or endorsed by the publisher.

## Abbreviations

PKD: paroxysmal kinesigenic dyskinesia
AED: antiepileptic drug
Vo: ventro-oral
Vim: ventral intermediate
DBS: deep brain stimulation.
